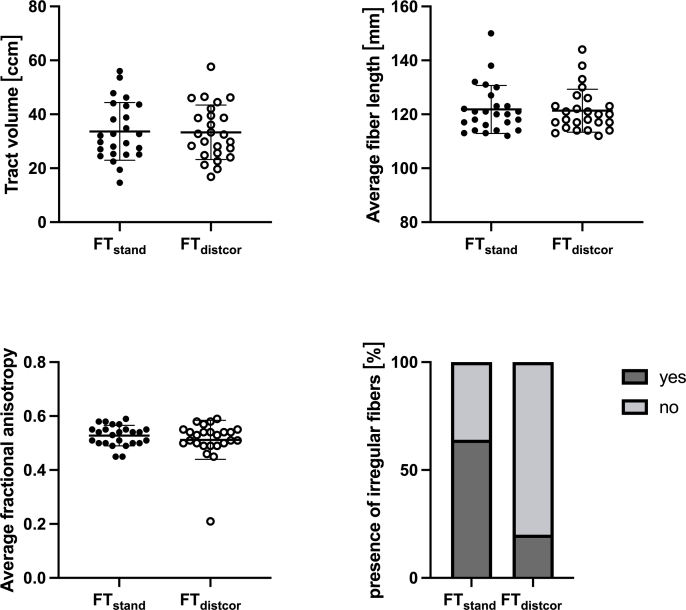# Improving tractography in brainstem cavernoma patients by distortion correction

**DOI:** 10.1016/j.bas.2023.102685

**Published:** 2023-10-06

**Authors:** Raimunde Liang, Maximilian Schwendner, Marc Grziwotz, Benedikt Wiestler, Maria Wostrack, Bernhard Meyer, Sandro M. Krieg, Sebastian Ille

**Affiliations:** a Department of Neurosurgery, Klinikum Rechts der Isar, Technische Universität München, Ismaninger Str. 22, 81675, Munich, Germany; b Department of Diagnostic and Interventional Neuroradiology, Klinikum Rechts der Isar, School of Medicine, Technische Universität München, Ismaninger Str. 22, 81675, Munich, Germany

**Keywords:** Fiber tracking, Brainstem cavernoma, Cranial distortion correction, Tractography

## Abstract

**Introduction:**

The resection of brainstem cerebral cavernous malformations (CCM) harbors the risk of damaging the corticospinal tract (CST) and other major tracts. Hence, visualization of eloquent fiber tracts supports pre- and intraoperative planning. However, diffusion tensor imaging fiber tracking at brainstem level suffers from distortion due to field inhomogeneities and eddy currents by steep diffusion gradients.

**Research question:**

This study aims to analyze the effect of distortion correction for CST tractography in brainstem CCM patients.

**Material and methods:**

25 patients who underwent resection of brainstem CCM were enrolled, 24 suffered from hemorrhage. We performed an anatomically based tractography of the CST with a mean minimal fractional anisotropy of 0.22 ± 0.04 before and after cranial distortion correction (CDC). Accuracy was measured by anatomical plausibility and aberrant fibers.

**Results:**

CDC led to a more precise CST tractography, further approximating its assumed anatomical localization in all cases. CDC resulted in a significantly more ventral location of the CST of 1.5 ± 0.6 mm (6.1 ± 2.7 mm before CDC vs. 4.6 ± 2.1 mm after CDC; p < .0001) as measured by the distance to the basilar artery and of 1.7 ± 0.6 mm (8.9 ± 2.7 mm vs. 7.2 ± 2.1 mm; p < .0001) in relation to the clivus. Aberrant fibers were reduced by CDC in 44% of cases. We found a mean difference in CST volume of 0.6 ± 0.8 ccm. We could not detect motor deficits after resection of irregular fibers.

**Discussion and conclusion:**

CDC effectively corrects tractography for distortion at brainstem level, especially in patients suffering from brainstem CCM, further approximating its actual anatomical localization.

## Highlights

• CDC improved the expected localization of CST by a more ventral positioning.
• Reduction of irregular fibers could be achieved through distortion correction.
• Semi-elastic fusion constitutes a readily applicable tool for clinical routine.

## Abbreviations

CCM: cerebral cavernous malformations
CDC: cranial distortion correction
CST: corticospinal tract
DTI FT: Diffusion tensor imaging fiber tracking
EPI: echo-planar imaging
FACT: fiber assignment by continuous tracking
FA: fractional anisotropy
FL: fiber length
FT: fiber tracking
MRI: magnetic resonance imaging
N: no
ROI: regions of interest
3D: three-dimensional

## 1 Introduction

Diffusion tensor imaging fiber tracking (DTI FT) has become a commonly used tool for pre- and intraoperative visualization of subcortical white matter pathways in neurosurgery (Bello et al., 2008; Prabhu et al., 2011; Mikuni et al., 2007; Maesawa et al., 2010). Especially when it comes to treat lesions in close vicinity to highly vulnerable structures, e. g. in brainstem surgery, DTI FT can facilitate treatment planning and risk stratification (Ulrich et al., 2014; Januszewski et al., 2016).

In order to make these data available during surgery, it is necessary to fuse DTI FT data with conventional anatomical, three-dimensional (3D) magnetic resonance imaging (MRI). This is usually done by rigid co-registration of 3D T1-weighted MRI and DTI data (Jeurissen et al., 2019). But due to the use of echo-planar imaging (EPI) in the acquisition of DTI, this type of data is especially prone to geometric distortions derived from static magnetic field (B0) inhomogeneities and motion artifacts. These artifacts come particularly into effect at air-tissue interfaces, e. g. in areas near the temporal petrous bone or at brainstem level (Wu et al., 2008; Zhang et al., 2020). This in turn can lead to spatial inaccuracies when the FT data is linearly projected on anatomical MRI data, because the geometric characteristics of anatomical structures may vary between the two datasets.

Since this is a well-known problem, several strategies have been proposed to correct EPI distortions using techniques like magnetic field mapping, reverse phase encoding or phase-shifted EPI pulse sequence acquisition (Jezzard and Balaban, 1995; Chen and Wyrwicz, 2001; Holland et al., 2010; Xiong et al., 2019). These methods rely on non-linear, deformable image co-registration and can be subsumed under elastic image fusion approaches. They can indeed improve the intra-modal image alignment very reliably, but also require additional scan time (Ruthotto et al., 2012). Furthermore, there are semi-elastic approaches available, using deformable models for nonrigid image co-registration (Huang et al., 2008; Liu et al., 2013; Gerhardt et al., 2019). In contrast to elastic image fusion approaches, they do not require an additional scan time, but often show a higher percentage of local image co-registration artifacts.

The aim of this study was to examine whether a semi-elastic fusion approach can be implemented into a clinical workflow, and whether this approach could improve the accuracy of DTI FT in brainstem cavernoma patients.

## 2 Material and methods

### 2.1 Ethics

The experimental setup was approved by our local ethics committee (registration number: 2793/10, 222/14, 336/17, 192/18, 18/19) and was conducted in accordance with the Declaration of Helsinki. Written informed consent was obtained from all patients prior to the examination.

### 2.2 Patients

Overall, we included 25 patients with brainstem cavernoma undergoing resection between 2008 and 2018 at our department. DTI sequences with at least 6 directions and a T1-weighted three-dimensional gradient echo sequence was available at least at one time point during surgical treatment in all cases. Patient characteristics and characteristics of all lesions were analyzed and patient outcome including motor status assessed in detail according to the Medical Research Council grading was acquired (Table 1).

**Table 1:** Patient characteristics Table 1 shows detailed patient characteristics of all included patients including the location of the cavernoma in relation to the brainstem and the incidence of preoperative hemorrhage in our cohort.

Number of cases	25
**Female gender (%)**	11 (44)
**Age at surgery (mean ± SD; y) (range)**	46.2 ± 18.8 (20–80)
**Location**	
• mesencephalon (%)	5 (20)
• pons (%)	5 (20)
• pons and mesencephalon (%)	1 (4)
• pons and medulla oblongata (%)	5 (20)
• cerebellum	9 (45)
**Previous hemorrhage (%)**	24 (96.0%)
**Preoperative DTI-Imaging**	16 (64)

### 2.3 Setup

#### 2.3.1 Distortion correction

DTI-derived B0 images were fused with conventional anatomical MRI data in all cases. Rigid image fusion was compared to semi-elastic image co-registration applying distortion correction (Elements Image Fusion Version 5.0, Brainlab AG, Munich, Germany).

After inter-modal rigid fusion of DTI and conventional anatomical MRI by a mutual information-based linear co-registration algorithm, distortion correction was used to elastically deform the geometrically distorted B0 images with respect to the reference scan. Image segmentation considering voxel-wise anatomic labeling of the 3D MRI was automatically performed using a synthetic tissue model, and the image volume related to the brain was subdivided into 3 × 3 × 3 cm³ image volumes. For semi-elastic image fusion, multiple affine co-registrations for each 3D subvolume were calculated, ultimately determining a 3D deformation vector field through interpolation of the local affine co-registration estimates. Here, the 3D deformation field described the pixel-wise morphing of the B0 image volume to achieve spatial alignment with the 3D reference MRI scan (Gerhardt et al., 2019).

#### 2.3.2 Anatomically based DTI FT

For anatomically based DTI FT we used our standard deterministic algorithm with fiber assignment by continuous tracking (FACT) (Brainlab AG, Munich, Germany). Preparation of the DTI data and computing of the white matter tracts was performed according to our standard protocol (Nimsky et al., 2006; Krieg et al., 2012).

We visualized the corticospinal tract (CST) twice in every patient, once using our standard technique (FT_stand_) and once after applying semi-elastic distortion correction (FT_distcor_). For each FT, two regions of interest (ROI) were defined: one ROI was located in the brainstem at tentorium level, while the second ROI was seeded over the primary motor cortex, which was identified by anatomical landmarks, e. g. the hand knob, on multiple axial slices creating a 3D volume of the precentral gyrus. The procedure was performed separately for each hemisphere. The minimum fiber length (FL_min_) and the maximum fiber angulation (Ang_max_) were predefined for every patient (FL_min_ = 100 mm, Ang_max_ = 20°). However, the minimum fractional anisotropy (FA) was individually defined for each patient during FT_stand_ and chosen for FT_distcor_ accordingly (FA = 0.22 ± 0.04, FA_max_ = 0.29, FA_min_ = 0.10). Finally, the CST identified by FT_stand_ and FT_distcor_ were included as an object in the 3D T1-anatomical MRI data set.

#### 2.3.3 Data analysis

To compare the different FT results, we analyzed the distance of the CST to predefined anatomical landmarks, namely the clivus and the basilar artery, as well as the absolute tract volume. Furthermore, we compared the two approaches in terms of the presence of irregular fibers, which were defined as fibers not connected to the main tract volume or with an inadequate fiber course, i. e. through the bone or CSF. Postoperative motor function was correlated to preoperative imaging for all patients who had preoperative DTI data (n = 16) by measuring the distance of the CST to the cavernoma. Finally, the different FT results were visually compared in relation to the expected anatomical location of the CST. Measurements were performed by a physician experienced in fiber tracking according to clinical routine. No intraobserver variability was described; interobserver variability was not assessed in detail – partially measurements performed by the operating surgeon were available, which showed high concordance.

Data analyses and descriptive statistics were conducted using GraphPad Prism (version 9.1.1 for Mac, GraphPad Software, La Jolla, CA, USA). All values are given in mean ± standard deviation (SD). A p-value of <.05 was considered statistically significant. Statistical differences of FT_stand_ and FT_distcor_ were assessed via Wilcoxon rank-sum tests.

## 3 Results

### 3.1 Fiber tracking analyses

**Fig. 1:**
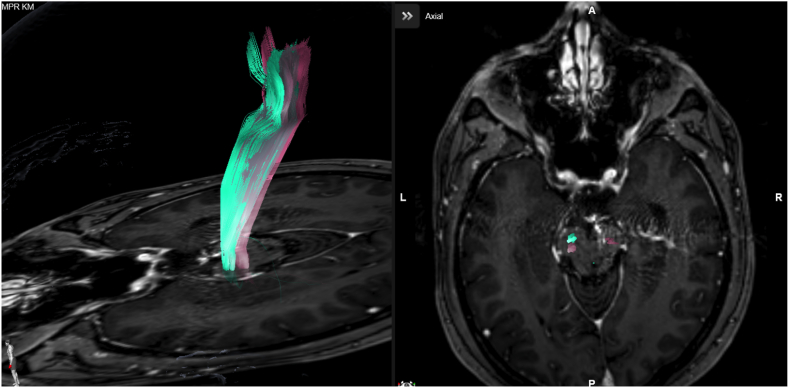
Fibertracking Fig. 1 shows an example of CST location before (red) and after (green) distortion correction on brainstem level. (For interpretation of the references to colour in this figure legend, the reader is referred to the Web version of this article.)

**Fig. 2:**
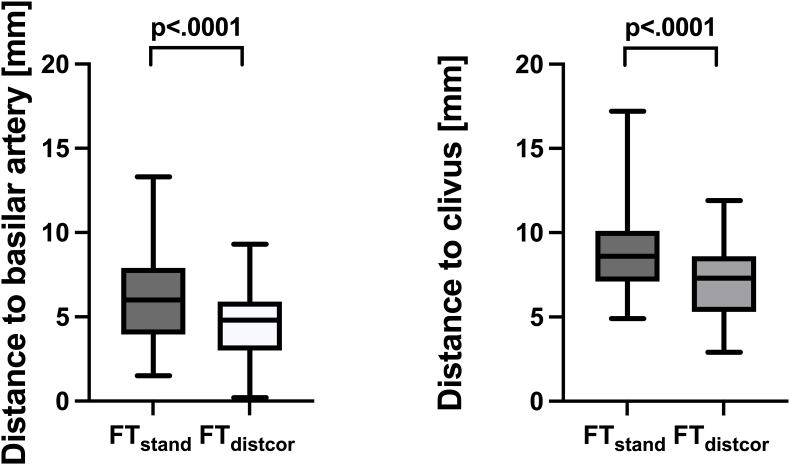
Measured distances to basilar artery and clivus Fig. 2 illustrates the distances between the CST and the basilar artery (left) and the distance between the CST and the clivus (right) for the standard technique (FT_stand_) and for semi-elastic distortion correction method before fibertracking (FT_distcor_).

We compared FT_stand_ and FT_distcor_ for each patient. We observed that FT_stand_ of the CST was generally located dorsal (n = 13) or dorsolateral (n = 12) of the expected anatomical location as assumed on anatomical imaging on brainstem level (Fig. 1, Supplementary Table 1). CDC resulted in a significantly more ventral location of the CST of 1.5 ± 0.6 mm (6.1 ± 2.7 mm before CDC vs. 4.6 ± 2.1 mm after CDC; p < .0001) as measured by the distance to the basilar artery and of 1.7 ± 0.6 mm (8.9 ± 2.7 mm vs. 7.2 ± 2.1 mm; p < .0001) as measured by the distance to the clivus (Fig. 2). Furthermore, significantly less cases showed irregular fibers with additional distortion correction compared to the standard technique (FT_distcor_ 20.0% vs FT_stand_ 64.0%, p < .001) (Table 3). The tract volume (V_CST_ 33.3 ccm vs 33.7 ccm for FT_distcor_ vs FT_stand,_ P = .37) as well as the average fiber length (FL_average_; 121 mm vs 122 mm for FT_distcor_ vs FT_stand_, P = .41) and the average fractional anisotropy (FA_average;_ 0.51 vs 0.53 for FT_distcor_ vs FT_stand_, P = .19) were not statistically significant different in the compared approaches (Supplementary Fig. 1).

### 3.2 Clinical correlation

We included 25 patients with cavernoma in our study, of which 24 suffered cavernoma related hemorrhage and were therefore operated on in a subacute setting. As exception to this, two patients were admitted with initial reduced vigilance and underwent surgery within 4 days for this reason. Median time span from date of diagnosis to surgery was 17 (2–259) days. One case underwent elective resection. In our cohort, 16 patients had preoperative DTI data, of which 2 patients (12.5%) showed a preoperative motor deficit which persisted postoperatively. Two patients showed new motor deficits postoperatively. Motor deficit in all four patients presented as a hemiparesis. The remaining nine patients underwent DTI imaging postoperatively and did not show motor deficits pre- or postoperatively. Ten patients presented cranial nerve deficits preoperatively, three of these ten patients showed an improvement postoperatively with persisting deficits remaining in four patients and aggravation of deficits in three patients. Two patients showed new cranial nerve deficits. Five patients showed sensory deficits, mainly hemihypesthesia, two patients reported an aggravation of sensory deficits while the remaining four patients reported no change (Table 2).

**Table 2:** Motor function The table shows the preoperative and postoperative motor function using the Medical Research Council grading for all patients with preoperative DTI. Furthermore, distances between the CST and the lesion for the standard technique (FT_stand_) and for semi-elastic distortion correction method before fibertracking (FT_distcor_) are shown.

Number of cases	25
**Preoperative motor deficits**	
**(number of patients (%))**	
• BMRC 5/5	23 (92)
• BMRC ± 4/5	2 (8)
**Postoperative motor deficits**	
**(number of patients (%))**	
• BMRC 5/5	21 (84)
• BMRC ± 4/5	4 (16)
**MRI imaging**	
**(number of patients (%) before/after CDC)**	
• Distance <1 mm	11 (68.8)/4 (25)
• Distance ≥1 mm < 2 mm	2 (12.5)/6 (37.5)
• Distance ≥2 mm	3 (18.8)/6 (37.5)

**Table 3:** Fibertracking before and after distortion correction The table compares the individual values of fibertracking data before and after distortion correction including tract volume, presence of irregular fibers and distance to the basilar artery and clivus as well as average fractional anisotropy (FA) and fiber length (FL).

	Tract volume (ccm)	Irregular fibers (cases(%))	Distance (cm)		Average FA	Average FL (mm)
			Basilar artery	clivus		
**FTstand**	33.7 ± 10.7	Y (64)	6.1 ± 2.7	8.9 ± 2.7	0.53 ± 0.04	127 ± 9
**FTdiscor**	33.3 ± 10.1	Y (20)	4.6 ± 2.1	7.2 ± 2.1	0.51 ± 0.07	117 ± 8

Distance of the core fiber volume to the CST did not correlate with the postoperative outcome regardless of additional distortion correction with a maximum distance of 0.9 mm without CDC and 2.4 mm with CDC in patients with postoperative motor deficits. However, we could detect more irregular fibers, which were partly running directly through the cavernoma or in its edge region, with the standard technique. Resection of these irregular fibers did not affect the postoperative outcome of the motor function in most cases, apparently (5/6 = 83%).

## 4 Discussion

The current study demonstrates that postprocessing of DTI data significantly improves the resulting tractography in the posterior fossa. While this might not have severe effects in supratentorial cases, the steep diffusion gradients by the petrous bone might even necessitate such postprocessing in the future as a standard procedure.

Microsurgical resection is regarded as main treatment for bled CCM (Zdunczyk et al., 2021). CCM located in the brainstem are prone to the risk of postoperative deficits due to the dense eloquent tissue of this area. With EPI being known to be susceptible against magnetic field inhomogeneities causing geometric distortion (Huang et al., 2008), distortion correction is particularly important in the case of brainstem cavernomas due to their location and the presence of surrounding structures and can improve the accuracy of the resulting tractography. Exact localization of the CST and other tracts provides valuable information for pre- and intraoperative planning as well as for risk stratification prior to surgery (Zdunczyk et al., 2021). Especially at brainstem level spatial inaccuracies of DTI-derived fiber tracts can be caused by susceptibility artifacts. Merhof et al. described an inaccuracy for the pyramidal tract in anterior-posterior direction at brainstem level with less affection of the left-right direction (Merhof et al., 2007). Correspondingly, in our cohort, we found that CST was generally dislocated in a posterior direction compared to anatomical imaging and located significantly more ventrally after distortion correction. Moreover, distortion correction was found to achieve a reduction of irregular fibers without affecting postoperative outcome, therefore increasing clinical relevance of the displayed tracts. In a clinical setting, a reliable preoperative identification of the relationship between the lesion and tracts facilitates safe resection due to proper approach selection and correct identification of safe zones.

In our series, motor function of two patients worsened postoperatively, which is in line with results of literature (Zdunczyk et al., 2021; Li et al., 2018). No significant relationship in the impairment of motor function and the distance of the core fiber volume to the cavernoma was detected regardless of CDC. Zdunczyk et al. described proximity of CST to the cavernoma <1 mm as a risk factor for a new postoperative motor deficit or worsening of motor function (Zdunczyk et al., 2021). It has to be noted, however, that the cohort of patients was small and validation in a bigger cohort is needed. Moreover, since the CST just served as a representative tract in this study, this might not be case for the proper visualization of other functional systems. For example, investigation of the corticobulbar tract regarding cranial nerve deficits could be added for future examinations. As mentioned beforehand, correction of EPI distortion can be achieved by two types of techniques. While a phase map or a field map offer robust correction of EPI distortions, these methods require more scan time and precise measurement which is much more challenging to apply to clinical routine whereas CDC can be easily integrated in the clinical workflow and preoperative surgical planning. Of note, CDC was feasible for all patients with retrospectively acquired data.

## 5 Limitations

Performed as a retrospective study in a small cohort of patients due to the rare prevalence of surgical resected brainstem CCM, CDC led to more ventral location of the CST as well as the reduction of irregular fibers suggestive for improvement of the accuracy. However, it has to be mentioned that MRI artifacts (e. g. edema and brainshift in case of hemorrhage) can lead to residual inaccuracies despite of distortion correction. Moreover, further refinement of the data can be achieved by a more recent imaging technique used for distortion correction based on high angular resolution diffusion (HARDI) signals, offering more robustness to motion and distortion artifacts and a higher reliability resolving complex fiber configurations. While both HARDI and DTI use similar correction techniques, HARDI constitutes a more versatile choice, especially regarding complex fiber anatomy. It is also known that semi-elastic fusion approaches are prone to co-registration artifacts (Gerhardt et al., 2019; Negwer et al., 2020) raising the question whether this approach is sufficient. A correlation of the clinical relevance using these techniques for further improvement is to be evaluated in the future. Intraoperative anatomical verification, e. g. by intraoperative monitoring and comparison with non-linear approaches remain to be investigated as the next step. Moreover, the present findings need to be validated in a larger cohort.

## 6 Conclusions

CDC is feasible for distortion correction at brainstem level in clinical routine, further approximating its anatomical localization. This information can improve accuracy for surgical planning and intraoperative image guidance and could provide a valuable tool for clinical routine enabling a more patient-specific approach in the future upon further validation.

## Disclosure

The study was financed from institutional grants. SK is consultants for Ulrich Medical (Ulm, Germany) and Need Inc. (Santa Monica, CA, USA); he received honoraria from Medtronic (Meerbusch, Germany), Nexstim Plc (Helsinki, Finland) and Carl Zeiss Meditec (Oberkochen, Germany). SI, SK and BM received research grants and are consultants for Brainlab AG (Munich, Germany). BM received honoraria, consulting fees, and research grants from Medtronic (Meerbusch, Germany), icotec ag (Altstätten, Switzerland), and Relievant Medsystems Inc., Sunnyvale, CA, USA), honoraria, and research grants from Ulrich Medical (Ulm, Germany), honoraria and consulting fees from Spineart Deutschland GmbH (Frankfurt, Germany) and DePuy Synthes (West Chester, PA, USA), and royalties from Spineart Deutschland GmbH (Frankfurt, Germany).

All authors declare that they have no conflict of interest regarding the materials used as well as the results presented in this study.

## Declaration of competing interest

All authors declare that they have no conflict of interest regarding the materials used as well as the results presented in this study.

## Figures and Tables

**figs1:**