# Supplementary material for: Improving tractography in brainstem cavernoma patients by distortion correction
Source: Brain Spine. 2023 Oct 6;3:102685. doi: 10.1016/j.bas.2023.102685 (PMC10668098; doi:10.1016/j.bas.2023.102685)
Supplement: Multimedia component 1 [file mmc1.docx]

| FTstand | | | | | | FTdistcor | | | | | |
| --- | --- | --- | --- | --- | --- | --- | --- | --- | --- | --- | --- |
| Tract volume (ccm) | Irregular fibers (Y/N) | Distance (mm) | | Average FA (mm) | Average FL (mm) | Tract volume (ccm) | Irregular fibers (Y/N) | Distance (mm) | | Average FA (mm) | Average FL (mm) |
|  |  | Basilar artery | clivus |  |  |  |  | Basilar artery | clivus |  |  |
| 43.6 | Y | 9.4 | 11.5 | 0.45 | 127 | 44.5 | Y | 5.7 | 9.0 | 0.46 | 127 |
| 38.1 | Y | 5.1 | 5.6 | 0.55 | 117 | 38.7 | N | 3.2 | 4.5 | 0.54 | 117 |
| 46.2 | Y | 7.0 | 9.1 | 0.51 | 114 | 46.0 | N | 3.5 | 6.6 | 0.51 | 114 |
| 34.8 | N | 7.9 | 8.7 | 0.50 | 121 | 36.1 | N | 4.5 | 8.5 | 0.50 | 121 |
| 44.1 | Y | 3.3 | 8.3 | 0.50 | 120 | 46.5 | Y | 2.6 | 5.4 | 0.50 | 120 |
| 53.6 | Y | 3.9 | 7.5 | 0.54 | 121 | 42.1 | N | 3.7 | 5.9 | 0.54 | 121 |
| 56.0 | N | 3.9 | 7.3 | 0.45 | 132 | 57.6 | N | 2.2 | 5.1 | 0.45 | 133 |
| 43.1 | N | 5.0 | 6.5 | 0.55 | 150 | 39.5 | N | 5.4 | 5.5 | 0.51 | 144 |
| 47.8 | Y | 6.0 | 8.5 | 0.55 | 130 | 46.2 | N | 3.8 | 8.5 | 0.55 | 130 |
| 29.3 | N | 8.4 | 12.5 | 0.50 | 122 | 29.8 | N | 6.1 | 10.1 | .0.49 | 122 |
| 25.3 | Y | 5.4 | 6.9 | 0.54 | 112 | 21.2 | N | 5.1 | 5.2 | 0.54 | 112 |
| 32.7 | Y | 6.7 | 10.5 | 0.49 | 131 | 32.8 | N | 6.6 | 7.6 | 0.49 | 126 |
| 38.8 | N | 6.4 | 9.6 | 0.51 | 138 | 38.6 | N | 5.6 | 8.0 | 0.21 | 138 |
| 27.0 | Y | 5.8 | 7.9 | 0.50 | 114 | 27.4 | N | 5.5 | 7.3 | 0.50 | 114 |
| 32.1 | Y | 1.5 | 6.9 | 0.53 | 113 | 33.3 | Y | 1.4 | 2.9 | 0.53 | 115 |
| 22.5 | Y | 7.9 | 9.7 | 0.54 | 118 | 22.6 | N | 6.3 | 8.2 | 0.54 | 118 |
| 27.5 | N | 6.4 | 5.3 | 0.55 | 118 | 28.1 | N | 4.8 | 5.0 | 0.55 | 118 |
| 32.8 | Y | 2.7 | 4.9 | 0.51 | 123 | 32.5 | N | 2.6 | 5.2 | 0.51 | 123 |
| 24.5 | Y | 10.3 | 11.2 | 0.49 | 123 | 25.6 | Y | 9.3 | 10.2 | 0.49 | 123 |
| 28.0 | N | 4.0 | 8.8 | 0.58 | 113 | 28.3 | N | 2.8 | 8.7 | 0.58 | 113 |
| 14.6 | Y | 13.3 | 17.2 | 0.58 | 121 | 16.8 | Y | 8.7 | 11.9 | 0.53 | 117 |
| 29.7 | N | 2.5 | 8.6 | 0.57 | 116 | 29.9 | N | 0.2 | 7.0 | 0.58 | 117 |
| 25.0 | Y | 6.1 | 8.7 | 0.59 | 117 | 24.8 | N | 4.8 | 8.4 | 0.59 | 117 |
| 19.4 | N | 5.0 | 12.6 | 0.57 | 114 | 19.7 | N | 4.0 | 8.7 | 0.57 | 114 |
| 25.1 | Y | 8.7 | 8.2 | 0.54 | 120 | 24.0 | N | 6.5 | 6.5 | 0.54 | 120 |

**Supplementary Table 1: Individual patient data before and after distortion correction**

The table compares the individual values of fibertracking data before and after distortion correction including tract volume, presence of irregular fibers and distance to the basilar artery and clivus as well as average FA und FL (Y = yes, N = no).
